# Uterine Artery Embolization of Uterine Arteriovenous Malformation: A Systematic Review of Success Rate, Complications, and Posterior Pregnancy Outcomes

**DOI:** 10.3390/jpm12071098

**Published:** 2022-07-01

**Authors:** Francisco Javier Ruiz Labarta, María Pilar Pintado Recarte, Manuel González Leyte, Coral Bravo Arribas, Arturo Álvarez Luque, Yolanda Cuñarro López, Cielo García-Montero, Oscar Fraile-Martinez, Miguel A. Ortega, Juan A. De León-Luis

**Affiliations:** 1 Department of Public and Maternal and Child Health, School of Medicine, Complutense University of Madrid, 28040 Madrid, Spain; javruila@hotmail.com (F.J.R.L.); ppintado@salud.madrid.org (M.P.P.R.); ycunarro@ucm.es (Y.C.L.); jaleon@ucm.es (J.A.D.L.-L.); 2 Maternal Fetal Medicine Research Group, School of Medicine, Complutense University of Madrid, 28040 Madrid, Spain; 3 Group of Pathophysiology in Women, Pregnancy, Labor, and Puerperium, Health Research Institute Gregorio Marañón, 28040 Madrid, Spain; manuelgonzalezleyte@gmail.com (M.G.L.); luque6000@gmail.com (A.Á.L.); 4 Maternal and Infant Research Investigation Unit, Alonso Family Foundation (UDIMIFFA), 28009 Madrid, Spain; 5 Department of Radiology, Division of Interventional Radiology, Hospital General Universitario Gregorio Marañón, Universidad Complutense de Madrid, 28040 Madrid, Spain; 6 Department of Medicine and Medical Specialities, Faculty of Medicine and Health Sciences, University of Alcalá, 28801 Alcala de Henares, Spain; cielo.gmontero@gmail.com (C.G.-M.); oscarfra.7@hotmail.com (O.F.-M.); 7 Ramón y Cajal Institute of Sanitary Research (IRYCIS), 28034 Madrid, Spain

**Keywords:** Uterine Arteriovenous Malformation (UAVM), hemorrhage, uterine artery embolization (UAE), systematic review

## Abstract

Uterine Arteriovenous Malformation (UAVM) is a rare but life-threating cause of uterine bleeding. The clinical management of this condition is challenging, and there is a need to describe the most adequate approach for these patients. Uterine artery embolization (UAE) is the most widely-published treatment in the literature in recent years, although there is a need to update the evidence on this treatment and to compare it with other available therapies. Thus, the objective of this systematic review is to quantify the efficacy of UAE of UAVM. In addition, we evaluated the clinical context of the patients included, the treatment complications, and the pregnancy outcomes after UAE. With this goal in mind, we finally included 371 patients spread over all continents who were included in 95 studies. Our results show that, similar to other medical therapies, the global success rate after embolization treatment was 88.4%, presenting a low risk of adverse outcomes (1.8%), even in women with later pregnancy (77% had no complications). To date, this is the largest systematic review conducted in this field, although there are still some points to address in future studies. The results obtained in our study should be outlined in UAE protocols and guidelines to aid in clinical decision-making in patients with UAVM.

## 1. Introduction

Uterine Arteriovenous Malformation (UAVM) is a rare but life-threating cause of uterine bleeding. UAVMs are defined as abnormal direct communications between arteries and veins without an intervening capillary network [1]. They can be congenital or acquired. Congenital cases are extremely rare and arise from an abnormal embryologic development in the primitive capillary plexus causing multiple connections between arteries and veins. However, acquired UAVMs are more common and typically consist of direct fistulas between intramural arterial branches and the myometrial venous plexus. They are caused by reactive angiogenesis, which occurs secondary to dilatation and curettage (D&C), uterine surgery, therapeutic abortion, gestational trophoblastic neoplasia, infection, direct trauma, and gynecological malignancies most commonly. In recent years, the incidence has increased due to increased uterine instrumentation and cesarean sections [2]. The International Society for the Study of Vascular Anomalies (ISSVA) classifies these lesions as high-flow vascular malformations [3].

Since its first description by Dubreuil and Loubat in 1926 [4], many clinical cases on UAVM have been published. The reported incidence is among patients who present with menorrhagia, so true incidence is unknown, but it is assumed to be low [5,6].

UAVMs should be suspected in women of childbearing age who present with abnormal vaginal bleeding and a negative b-HCG (differential diagnosis with retained products of conception and gestational trophoblastic disease). UAVMs must be accurately diagnosed because uterine instrumentation may damage vessels that extend to the endometrium and result in catastrophic hemorrhage. Historically, UAVMs were diagnosed by laparotomy or pathologically after hysterectomy. Nowadays, ultrasound is often the first imaging examination performed because it is accessible and non-invasive. Color Doppler Imaging demonstrates multidirectional, high-velocity arterial flow within the myometrium. Computed tomography and magnetic resonance imaging (MRI) can be useful for treatment planning. The final diagnosis of UAVM is confirmed by angiography, which is the “gold standard” technique [7]. Early venous contrast filling of a uterine vascular network is the pathognomonic finding.

There is a paucity of high-level evidence guiding clinicians with respect to UAVM management. It depends on patient symptomatology, hemodynamic state, size and location of the lesions, and age, as well as the desire for future fertility. Sometimes a blood transfusion and utero-cervical-vaginal tamponade for severe vaginal bleeding is necessary. Medical treatments include: progestins, gonadotropin-releasing hormone agonists (GnRH-a), methotrexate, combined hormonal contraception, uterotonics, or danazol [8]. However, uterine artery embolization (UAE) is the most widely-published treatment in the literature in recent years. It has become a well-recognized minimal invasion alternative to surgical intervention for UAVMs, with the major advantage of maintaining childbearing capacity. Besides, it is fast, with minimal side effects and complications, shorter hospital stays, and a faster recovery.

The first description of a successful embolization treatment for UAVM was reported in 1986 [9]. Subsequently, multiple case reports and case series have been published, being very heterogeneous in terms of clinical context, efficacy, and technical characteristics. The last systematic review of acquired UAVM treated by EAP included studies published between 2003 and 2013. It met 40 studies comprising of 54 patients with a success rate with symptomatic control of 61% after first embolization [10]. With our study, we want to systematically review the literature on the management of UAVMs with UAE to update the evidence on this treatment and to compare it with other proposed managements, such as the medical treatment recently reviewed in a 2021 publication [8].

Our objective was to synthesize the efficacy data on the management of UAVMs with UAE. Furthermore, we aimed to evaluate UAVM etiology event of patients included and the treatment complications and pregnancy outcomes after UAE. Finally, we analyzed the factors associated with treatment success to aid in clinical decisions.

## 2. Material and Methods

This study was registered with the PROSPERO database (protocol CRD42021269510) and completed by conforming to the PRISMA 2020 guideline for systematic reviews and meta-analysis [11]. As this was a systematic review, no research ethics board review was required.

### 2.1. Information Sources and Literature Search

We performed an electronic database search of MEDLINE and Cochrane from 1 January 2000 to 18 September 2021 for full-text articles and published abstracts. Utilizing combinations of the relevant medical subject heading (MeSH) terms, keywords, and word variants, the condition of “UAVM” was combined with “UAE” (the compressive search strategy is presented in Supplementary S1). The search was limited to humans, and we did not limit the search by language, geographic origin, or study type. The titles and abstracts were screened to identify relevant articles. Three authors reviewed all the abstracts independently for eligibility, assessed the risk of bias, and extracted the data. Inconsistencies were discussed by the reviewers and a consensus was reached between them or by discussion with a fourth author (L.L.). Reference lists of relevant articles and reviews were hand-searched for additional reports that meet the inclusion criteria.

### 2.2. Elegibility Criteria

We included all studies—independent of their study design (case report, case series, observational or randomized interventional studies)—that described the treatment of UAVM with UAE. Only studies reporting the diagnosis of UAVM by angiography were considered suitable for inclusion. Only full-text articles were considered eligible for inclusion. Articles were excluded if medical or surgical management was concurrently initiated with UAE to treat UAVMs. Duplicates, letters-to-the-editor, editorials, and review articles were excluded. Furthermore, studies published before 2000 were not included, as advances in the technology of UAE make them less relevant.

### 2.3. Outcome Measures, Data Collection, and Risk of Bias Assessment

The primary outcome explored in the present systematic review was the success rate of UAE of UAVM. We differentiated primary UAE success rate with symptomatic control after first embolization (defined as complete hemorrhage arrest with hemodynamic stabilization and no subsequent medical or surgical procedure within the study’s follow-up period) and secondary UAE success rate after repeated embolization. UAVM may require repeat embolization for treatment, and this should not be considered as a failure of embolization, but rather as a requirement for additional treatments. The secondary outcomes were: UAVM etiology event (defined as associated clinical history for reported cases of UAVM) and patient UAE-related complications. Finally, we explored the pregnancy outcomes after UAE when reported.

A data extraction sheet was completed with the variables studied: author; publication year; country; study design; number of patients; age; gravity; parity; etiology event; time of diagnosis; symptoms; method of diagnosis; vessel embolized; laterality of embolization; embolic material utilized; success rate; requirement for additional embolization procedure, medical management, or surgery; transfusion; complications; and subsequent pregnancy. Data were extracted independently by three authors, who included them in the extraction sheet. Discrepancies were resolved by authors checking the study against the form.

A quality assessment of the included studies was performed using a customized version of a previously published framework to evaluate the methodologic quality of non-comparative studies, such as case reports and case series [12]. Each study is judged on four domains: selection, ascertainment, causality, and reporting, with a maximum total score of 7 (Supplementary S2).

### 2.4. Statistical Analysis

A statistical analysis was performed using SPSS Version 21.0 (IBM Corp.) in its default settings. The results were expressed as rates (%) for dichotomous variables and means for continuous variables. Ninety-five percent confidence intervals (95% CI) were calculated for the UAE success rate.

Given the heterogeneity of the clinical data of the included studies, a multivariate analysis could not be performed.

## 3. Results

A total of 246 articles were identified through electronic database searching and assessed with respect to their eligibility for inclusion. A hundred and fifty-eight articles had the full text assessed for eligibility and, finally, after exclusion for various reasons, 95 studies were included in the systematic review (Supplementary S3 for PRISMA diagram).

The included studies were composed of 82 case report studies and 13 case series studies representing data for 371 patients who underwent UAE for UAVM. The case report studies included a total of 124 patients and the case series studies had a total of 247 patients (range of 5–62 patients).

All the authors were different, except for two of them who repeated publications with different patients. Within the study period (January 2000 to September 2021), an increase in the number of articles published from 2013 was reduced, finding 59 articles (62.1% of the total) from that date. We found articles published throughout the world, with Asia being the continent with the largest number of articles (44 articles with 233 patients (62.8%)), followed by North America (23 articles with 61 patients (16.4%)) and Europe (18 articles with 66 patients (17.8%)).

Regarding the clinical characteristics of the patients included, the mean age was 31.3 years (range: 16–60 years), with 99% under 50 years. The mean of gravity was 1.99 (data collected from 331 patients) and the mean of parity was 1.19 (data collected from 233 patients). Almost all the articles did not specify data on the use of fertility treatment. Regarding the associated clinical history for reported cases of UAVM (Table 1), 91.6% of the patients had a history of an obstetric event. Forty-eight percent (178 patients) were associated with a previous abortion (most treated by curettage). Around eighteen percent (68 patients) were associated with unspecified obstetric manipulations. About twelve percent (47 patients) of the UAVMs were discovered postpartum (vaginal or cesarean section). As well, the less frequent causes of UAVM were associated with gestational trophoblastic disease (39 patients, 10.5%), gynecological problems (31 patients, 8.4%), and ectopic pregnancy (7 patients, 1.9%). There was a case of UAVM treated by embolization at 20 weeks of gestation in a patient with 2 previous cesarean sections, wherein the antepartum course was complicated by late-onset intrauterine growth restriction, but a healthy baby was born without sequelae. The median time between the etiology event and the development of AVM or vaginal bleeding was 42 days (data collected from 262 patients), with a range between 0 days and 15 years. The vast majority of UAVM gave symptoms in the form of persistent and uncontrollable profuse vaginal bleeding, documenting only five cases of asymptomatic patients with a casual finding of UAVM.

We did not find any demographic variable that was significantly associated with treatment success.

All UAVMs were finally diagnosed by angiography (according to the inclusion criteria of the systematic review), although ultrasound was the main initial imaging test in the diagnostic process. Ultrasound was obtained alone in 260 patients (70% of the total) and ultrasound together with another imaging test (MRI or CT) was conducted in 99 patients. There were three cases of UAVM diagnosed by hysteroscopy. The most frequent ultrasound description of UAVM that appears in the studies is in the form of an/hypoechoic tortuous spaces involving the uterine wall and with Doppler application, which demonstrated numerous dilated and tortuous blood vessels with a typical multidirectional high velocity and low resistance flow in the myometrium.

The uterine artery was the main embolized vessel (308 patients, 83%). There were four cases of uterine and ovarian artery embolization and one case of internal iliac artery (in 58 patients the embolized vessel was not specified). Embolization was performed bilaterally in 241 patients (65%) and laterality was not specified in 52 patients. In 52.3% of the patients, a mixture of materials was used for embolization. As the only embolizing agent, the most used was polyvinyl alcohol particles (13.7%) (Table 2).

**Figure 1:**
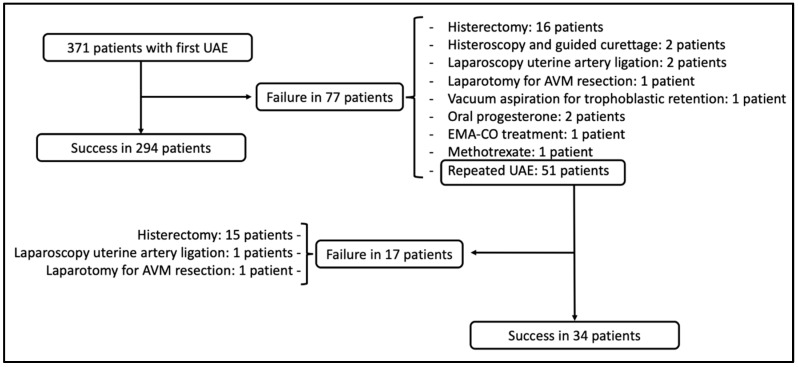
Flowchart to describe posterior management of first embolization procedure failure cases.

The primary UAE success rate with symptomatic control after first embolization was 79.2% and the secondary UAE success rate after repeated embolization was 66.7%. The global success rate after embolization treatment was 88.4% (Table 3). There were 43 patients who required additional medical management or surgery after failed embolization, with hysterectomy being the definitive treatment in 72% of these patients (Table 4 and Figure 1).

A temporal and geographic analysis of success rates was performed. It was found that the primary success rate after the first embolization in the articles published after 2010 (81.5%) was significantly higher than that of the articles published before 2010 (69.6%) (*p* 0.028)—without finding significant differences in the rate of secondary success after repeated embolization. No statistically significant differences were found in the success rates of the procedure between the different continents in which the articles were published (Table 5).

Fifty-eight patients (15.6% of the total) who presented minor complications in the form of pelvic or abdominal pain +/− fever, referred to by many authors as postembolization syndrome, were registered. There were six cases (1.6%) of major complications (Table 6), which highlighted three cases of pulmonary embolism. Seventy-six patients (20.5%) who required a transfusion of packed red blood cells with an average of 3.5 bags of blood per patient were documented, but this data was not correctly detailed by many authors. No case of death was recorded. The follow-up of the patients included in the study ranged from 3 days to 13 years.

Among the studies that collected data on subsequent pregnancy, 77 patients (20.7% of the total number of patients included in the review) who had a pregnancy after UAE for UAVM were found. The average time interval between embolization and pregnancy outcome varied between 2 months and 5 years. Eighteen pregnancy complications were reported: 1 ectopic pregnancy, 6 miscarriages, 7 elective abortions, 1 premature labor at 24 weeks gestation where the newborn died 1 week later, 1 APP 24 weeks, 1 fetal growth restriction, and 1 term vaginal postpartum hemorrhage. Cesarean delivery was recorded in 10 patients. No AVMs recurred in pregnancy or postpartum.

Upon risk of bias assessment, the average score of publications included was 4.6 of a possible 7 points, with the case series studies having an average score (5.2) that was slightly higher than the case report studies (4.5). Studies were often downgraded for selection and reporting domains; they lacked details on how they selected patients for the intervention and treatment details to allow for replications. See Supplementary S2 for a summary of the risk of bias scores by study.

## 4. Discussion

### 4.1. Main Findings

In this systematic review of 95 studies, we evaluated data for 371 patients who received UAE for treatment of UAVM. The global success rate after embolization treatment was 88.4%, with 79.2% after first embolization and 66.7% after repeated embolization. As well, 91.6% of the patients with UAVM had a history of an obstetric event, the most frequent being a history of abortion (48% of the patients). Only 1.6% of the patients presented major complications associated with UAE, especially in the form of pulmonary embolism. Finally, 77 patients (20.7%) who had a pregnancy after UAE for UAVM were collected, 77% of whom had no complications and there was no recurrence of UAVM in any case.

The profile of the patient who underwent UAE by UAVM was that of a young patient (31 years old) of reproductive age with a mean parity of 1.19. These data highlight that embolization is a treatment that preserves fertility and, therefore, is especially indicated in young patients who may have a future reproductive desire.

The primary UAE success rate after first embolization (79.2%) described in our study is above the rate of 61% provided by the previous systematic review that included patients from 2003 to 2013 [10]. We have verified that in the articles published in the last 10 years, a significant improvement in success has been observed compared to the previous period (*p* < 0.05), which could be related to an improvement in technical equipment, embolizing material, and the increased experience of professionals performing this treatment more frequently in recent years. The fact that the success rate after the first embolization is higher than after repeated embolizations (79.2% vs. 66.7%) indicates that the longer it takes to correct the bleeding, the less successful the embolization is. The time factor has been associated with the failure of other obstetric hemorrhage treatments, such as the Bakri balloon [13]. There have been no studies comparing the effectiveness of repeat embolization versus medical therapy or hysterectomy for persistent bleeding after initial embolization. Clinical factors that predict the success or failure of embolization should be sought, but the studies published to date provide heterogeneous data that are very difficult to systematize in order to carry out this analysis.

In 2021, a systematic review on the medical treatment of UAVM (32 studies, 121 women) was published, presenting an overall success rate of 88%—a figure similar to that obtained in our systematic review with embolization [8]. This review included studies with a definition of UAVM that relied on the investigators’ individual description and a transfusion rate of 2.5% after initiation of medical therapy. However, in our work, the included studies were selected after the gold standard diagnosis of UAVM by angiography and there was a 20% transfusion rate. With these data, it is confirmed that the clinical severity of the patients and their heterogeneous selection mean that they are not comparable populations. Although both treatments are a fertility-sparing option, medical treatment would be useful in stable patients with minimal bleeding, while UAE remains the treatment of choice in patients who are hemodynamically unstable or have significant uterine bleeding. The vast majority of patients with UAVM included in our review had a more-or-less recent obstetric history, with abortion treated by curettage being the most common. This is clearly in line with what has already been published in previous systematic reviews [10,14]. Some authors comment that hormonal changes associated with pregnancy may play a role in the proliferation of latent UAVMs through an unknown mechanism [10,15], but the variables that would allow us to identify patients with a higher risk of developing a UAVM, to perform an early and close follow-up to avoid the associated complications, are unknown. The general findings of our review indicate that in the event of abnormal uterine bleeding in a patient with a recent diagnosis of abortion or another obstetric event, we should rule out UAVM from among the possible diagnoses. Given that they have different management and treatment procedures, it is essential to make a differential diagnosis between acquired UAVM (currently called by many authors as enhanced myometrial vascularity (EMV) [16]) and retained products of conception (RPOC). For this reason, works are emerging to help differentiate it by color Doppler ultrasound [17], representing a non-invasive means of diagnosis and determination of management. However, most authors continue to consider angiography the gold-standard method for the diagnosis of UAVM and, therefore, we use this criterion to carry out the bibliographic search.

Despite the fact that EMV is the term used recently to describe acquired UAVM, most authors continue to refer to the UAVM name in their works. For this reason, we believe that not including this term in our bibliographic search could have minimally affected the collection of articles.

Thanks to the data collected in our review that confirm a rate of major complications of UAE of 1.6%, it can be stated that UAE is a very safe treatment for patients with UAVM. Other systematic reviews [18,19] analyzing the complications of UAE used as a treatment for other pathologies, such as postpartum hemorrhage, agree with the high safety of this treatment.

Despite the fact that many authors included in our systematic review did not document data on fertility after UAE for UAVM, we managed to collect 77 patients with documented pregnancy after UAE. Seventy-seven percent of pregnancies were uneventful, and elective or spontaneous abortion was the most frequently described gestational complication. The ischemic injury of the endometrium caused by UAE may play a role in subsequent pregnancy loss, although more studies would be necessary to confirm this association. Previous works have related UAE with an increased risk for placental abnormalities in subsequent gestations. Ref. [20] (specifically placenta accreta) does not seem to be confirm the data collected in our work. Soro et al. [21] indicated that UAE has no direct effect on the placental blood supply and fetal growth because the formation of collateral circulation develops very quickly after UAE. There are some studies suggesting a higher rate of infertility, premature ovarian failure, and uterine synechia [22], but they present heterogeneous data and a small number of patients, so this association has a very low degree of evidence.

### 4.2. Strengths and Limitations

Our systematic review includes the largest sample of patients with UAVM treated by UAE to date with 95 studies (published from 2000 to 2021) involving 371 patients spread over all continents. Yoon et al. [10] published a systematic review on this topic that included 40 studies (published from 2003 to 2013) with 54 patients. In our work, 62% of the included studies were published after 2013, which reflects the growing use and interest in this treatment in the literature and the need to update the data given the striking increase in articles in the literature. The greater number of patients in our review has allowed us to carry out a temporal and geographical analysis of the success rate of UAE, which has not been carried out in the literature to date. In the previous review, Yoon et al. performed a unilateral/bilateral analysis of embolization and embolic agents used and concluded that the published data is very heterogeneous (in terms of numerous combinations and options in embolic agent utilized, UAVM size, and clinical symptoms) and there were no significant differences in the necessity for repeat embolization. Nowadays, no controlled studies have been performed to evaluate the effectiveness of a unilateral versus bilateral treatment approach or comparing the efficacy of each embolic agent or combination of agents.

Given the rarity of UAVM, most of the studies identified were retrospective case report and case series studies of a few patients with mostly positive outcomes. Publication bias is very likely because small studies with negative results are often not published. The data included in the articles is very heterogeneous in terms of clinical scenario, embolic agent used, and unilateral or bilateral approach, making it impossible to carry out comparison studies to evaluate the effectiveness of different treatment approaches. In addition, the case series provide global data that prevent a detailed study of the cases. Although more evidence is needed on this topic with studies of higher methodological quality, the rarity of UAVM leaves study options limited, and reports such as ours are likely to be the highest available evidence from which to draw guidance for clinical decision making.

## 5. Conclusions

Our systematic review supports the effectiveness of UAE in the clinical management of UAVM with a high global success rate (88.4%), demonstrating its safety with a very low risk of complications (1.8%). Also, we observed that this procedure could also be recommended in women with later pregnancy (77% of them did not present any adverse outcomes during this period and, in any case, there was UAVM recurrence), although further studies are required in this group. These data are due to the improvement in technical equipment, embolizing material, and an augmented experience of professionals performing this therapeutic approach. To date, this is the largest systematic review conducted in this field, however there are some issues to address that are derived from the high heterogeneity of the available studies—in terms of clinical scenario, embolic agent used, or unilateral versus bilateral approach. However, due to the difficulties and rarity of UAVM, we consider that the results obtained in our study should be outlined in UAE protocols and guidelines to aid in clinical decision-making.

## Figures and Tables

**Table 1:** Clinical history for reported cases of UAVM.

UAVM Etiology Event	N (%)
**Abortion**	**178 (48%)**
* Curettage	111
* Medical or spontaneous	19
* Not specified	48
**Obstetric manipulations (not specified)**	**68 (18.3%)**
**Postpartum**	**47 (12.7%)**
* Vaginal	20
* Cesarean section (one uterine scar defect)	21
* Curettage	2
* Artificial removal of placenta	4
**Gestational trofoblastic disease (GTD)**	**39 (10.5%)**
**Gynecological problems**	**31 (8.4%)**
* Gynecological bleeding	20
* Placement of IUD (one uterine perforation)	3
* Laparoscopic or hysteroscopic procedures: 3 myomectomy, 1 polyp resection	6
* Laparotomic procedures: 1 ovarian endometrioma, 1 total hysterectomy	2
**Ectopic pregnancy**	**7 (1.9%)**
* Cervical	1
* Cesarean scar (one heterotopic)	4
* Intersticial/Tubaric	2
**Gestation in course**	**1 (0.3%)**
**Total**	**371 (100%)**

**Table 2:** Embolic material utilized in the studies included.

Embolic Material Utilized	N (%)
Mixed	194 (52.3%)
PVA	51 (13.7%)
Liquid agent	35 (9.4%)
HGS	32 (8.6%)
Microspheres	15 (4%)
Coils	11 (3%)
N.R.	33 (9%)
Total	371 (100%)

**Table 3:** Global success rate after first and repeated embolization treatment.

	Positive UAE	Total Patients	Success Rate
Primary success rate with symptomatic control after first embolization	294	371	79.2%
Secondary success rate after repeated embolization	34	51	66.7%
Global success rate after embolization treatment	328	371	88.4%

**Table 4:** Additional medical management or surgery required in the studies included.

Requeriment for Additional Medical Management or Surgery	N (%)
Histerectomy	31 (72.1%)
Histeroscopy and curettage	2 (4.6%)
Methotrexate	1 (2.3%)
Laparotomy for resection of AVM	2 (4.6%)
Laparoscopic uterine ligation	3 (7%)
Oral progesterone	2 (4.6%)
Vacuum aspiration for trophoblastic retention	1 (2.3%)
6 cycles of EMA-CO	1 (2.3%)
TOTAL	43

**Table 5:** Differences in primary, secondary, and global success rates prior to and after 2010 and across continents.

	<2010	≥2010	Asia	North America	Europe
Primary success rate	69.6% (48/69)	81.5% (246/302)	78.1% (182/233)	75.4% (46/61)	86.4% (57/66)
Secondary success rate	71.4% (10/14)	64.9% (24/37)	63.3% (19/30)	75% (9/12)	62.5% (5/8)
Global success rate	84.1% (58/69)	89.4% (270/302)	86.3% (201/233)	90.2% (55/61)	93.9% (62/66)

**Table 6:** Complications reported after embolization treatment.

One Disseminated Intravascular Coagulopathy (DIC)	Hospitalized in ICU.
One Uterine artery rupture during wire manipulation for embolization	
One non-flow limiting dissection of the internal iliac artery	
One Pulmonary embolism	Low blood oxygen saturation after UAE. She underwent an urgent tracheal intubation and mechanical ventilation for 2 days until the blood oxygen saturation returned to normal.
One Pulmonary Glue embolism	She developed mild chest discomfort after the injection of glue. She was tachypneic but maintained 100% saturation on room air. She was started on low molecular weight heparin (LMWH). Chest X-ray showed cardiomegaly with prominent central pulmonary vasculature and branching radio-opacities in bilateral lung fields (features suggestive of particulate embolism). Two-dimensional echocardiography showed right ventricular dysfunction with severe tricuspid regurgitation and severe pulmonary arterial hypertension. Computed tomography pulmonary angiography revealed multiple hyper-dense filling defects in segmental branches of the right upper lobe and subsegmental branches of the right and left pulmonary arteries secondary to glue embolism. LMWH was stopped subsequently as the patient improved clinically.
One Pulmonary embolism with cardiac arrest	2 h after the UAE the first attack of pulmonary embolism occurred, which was treated by anticoagulation therapy. She had cardiac arrest without palpable pulses, and got cardiopulmonary resuscitation (CPR) for 4 min. After achievement of normal cardiac activity, she was hemodynamically unstable. The subsequent echocardiography (ECG) revealed right atrium and ventricle enlargement, moderate tricuspid insufficiency, and inferior vena cava dilatation with elements of spontaneous echo contrasting. Extracorporeal membrane oxygenation was initiated and the patient was transferred to the ICU. Second attack happened on the third post-interventional day. Considering vaginal bleeding, continued extracorporeal membrane oxygenation (ECMO) and suspicion of embolic particles arising from uterus, a subtotal hysterectomy was done. The patient stayed in the ICU for 5 days, until systemic and hemodynamic stabilization. On the 11th day, she recovered completely.

## Data Availability

The datasets used and/or analyzed during the present study are available from the corresponding author on reasonable request.

## Supplementary Materials

The following supporting information can be downloaded at: https://www.mdpi.com/article/10.3390/jpm12071098/s1, Supplementary S1: Search Strategy; Supplementary S2: TITTLE; Supplementary S3: PRISMA Flow Diagram.
