# Supplementary material for: Uterine Artery Embolization of Uterine Arteriovenous Malformation: A Systematic Review of Success Rate, Complications, and Posterior Pregnancy Outcomes
Source: J Pers Med. 2022 Jul 1;12(7):1098. doi: 10.3390/jpm12071098 (PMC9324499; doi:10.3390/jpm12071098)

Supplementary S1: Search Strategy

Database: Ovid MEDLINE(R) ALL. Search Strategy:

----

1 Uterus/ 164,707
2 Uterine Artery/ 9,151
3 Uterine Hemorrhage/ 25,640
4 arteriovenous malformation/ 37,586
5 arteriovenous fistula/ 25,607
6 AVM/ 6,002
7 1 or 2 or 3 (187,956)
8 4 or 5 or 6 (46,275)
9 7 and 8 (474)

10 Embolization/ 178,650
11 Pelvic arterial embolization/ 1,912
12 Uterine artery embolization/ 2,607
13 UAE/ 10,148
14 transcatheter embolization/ 8,752
15 TCE/ 3,779
16 10 or 11 or 12 or 13 or 14 or 15 (191,575)
17 9 and 16 (278)
18. from 2000/1/1 - 2021/9/18 (246)

Search number	Query	Sort By	Filters	Search Details	Results	Time
18	#9 AND #16		from 2000/1/1 - 2021/9/18	(("uterus"[MeSH Terms] OR "uterus"[All Fields] OR "uteri"[All Fields] OR ("uterine artery"[MeSH Terms] OR ("uterine"[All Fields] AND "artery"[All Fields]) OR "uterine artery"[All Fields]) OR ("uterine haemorrhage"[All Fields] OR "uterine hemorrhage"[MeSH Terms] OR ("uterine"[All Fields] AND "hemorrhage"[All Fields]) OR "uterine hemorrhage"[All Fields])) AND ("arteriovenous malformations"[MeSH Terms] OR ("arteriovenous"[All Fields] AND "malformations"[All Fields]) OR "arteriovenous malformations"[All Fields] OR ("arteriovenous"[All Fields] AND "malformation"[All Fields]) OR "arteriovenous malformation"[All Fields] OR ("arteriovenous fistula"[MeSH Terms] OR ("arteriovenous"[All Fields] AND "fistula"[All Fields]) OR "arteriovenous fistula"[All Fields]) OR "AVM"[All Fields]) AND ("embol"[All Fields] OR "embolics"[All Fields] OR "embolisations"[All Fields] OR "embolise"[All Fields] OR "embolised"[All Fields] OR "embolising"[All Fields] OR "embolism"[MeSH Terms] OR "embolism"[All Fields] OR "embolic"[All Fields] OR "embolisms"[All Fields] OR "embolization, therapeutic"[MeSH Terms] OR ("embolization"[All Fields] AND "therapeutic"[All Fields]) OR "therapeutic embolization"[All Fields] OR "embolisation"[All Fields] OR "embolization"[All Fields] OR "embolizations"[All Fields] OR "embolize"[All Fields] OR	246	04:05:54

				"embolized"[All Fields] OR "embolizes"[All Fields] OR "embolizing"[All Fields] OR (("pelvics"[All Fields] OR "pelvis"[MeSH Terms] OR "pelvis"[All Fields] OR "pelvic"[All Fields]) AND ("arterialization"[All Fields] OR "arterializations"[All Fields] OR "arterialize"[All Fields] OR "arterialized"[All Fields] OR "arterializing"[All Fields] OR "arterially"[All Fields] OR "arterials"[All Fields] OR "arterie"[All Fields] OR "arteries"[MeSH Terms] OR "arteries"[All Fields] OR "arterial"[All Fields] OR "arteris"[All Fields] OR "artery"[All Fields] OR "arterious"[All Fields] OR "artery s"[All Fields] OR "arterys"[All Fields]) AND ("embol"[All Fields] OR "embolics"[All Fields] OR "embolisations"[All Fields] OR "embolise"[All Fields] OR "embolised"[All Fields] OR "embolising"[All Fields] OR "embolism"[MeSH Terms] OR "embolism"[All Fields] OR "embolic"[All Fields] OR "embolisms"[All Fields] OR "embolization, therapeutic"[MeSH Terms] OR ("embolization"[All Fields] AND "therapeutic"[All Fields]) OR "therapeutic embolization"[All Fields] OR "embolisation"[All Fields] OR "embolization"[All Fields] OR "embolizations"[All Fields] OR "embolize"[All Fields] OR "embolized"[All Fields] OR "embolizes"[All Fields] OR "embolizing"[All Fields])) OR ("uterine artery embolization"[MeSH Terms] OR ("uterine"[All Fields] AND "artery"[All Fields] AND "embolization"[All Fields]) OR "uterine artery embolization"[All Fields]) OR "UAE"[All Fields] OR ("transcatheter"[All Fields] AND ("embol"[All Fields] OR "embolics"[All Fields] OR "embolisations"[All Fields] OR "embolise"[All Fields] OR "embolised"[All Fields] OR "embolising"[All Fields] OR "embolism"[MeSH Terms] OR "embolism"[All Fields] OR "embolic"[All Fields] OR "embolisms"[All Fields] OR "embolization, therapeutic"[MeSH Terms] OR ("embolization"[All Fields] AND "therapeutic"[All Fields]) OR "therapeutic embolization"[All Fields] OR "embolisation"[All Fields] OR "embolization"[All Fields] OR "embolizations"[All Fields] OR "embolize"[All Fields] OR "embolized"[All Fields] OR "embolizes"[All Fields] OR "embolizing"[All Fields])) OR ("chem educ"[Journal] OR "tce"[All Fields]))) AND (2000/1/1:2021/9/18[pdat])		
**17**	#9 AND #16			("uterus"[MeSH Terms] OR "uterus"[All Fields] OR "uteri"[All Fields] OR ("uterine artery"[MeSH Terms] OR ("uterine"[All Fields] AND "artery"[All Fields]) OR "uterine artery"[All Fields]) OR ("uterine haemorrhage"[All Fields] OR "uterine hemorrhage"[MeSH Terms] OR ("uterine"[All Fields] AND "hemorrhage"[All Fields]) OR "uterine hemorrhage"[All Fields])) AND ("arteriovenous malformations"[MeSH Terms] OR ("arteriovenous"[All Fields] AND "malformations"[All Fields]) OR "arteriovenous malformations"[All Fields] OR ("arteriovenous"[All Fields] AND "malformation"[All Fields]) OR "arteriovenous malformation"[All Fields] OR ("arteriovenous fistula"[MeSH Terms] OR ("arteriovenous"[All Fields] AND "fistula"[All Fields]) OR "arteriovenous fistula"[All Fields]) OR "AVM"[All Fields]) AND ("embol"[All Fields] OR "embolics"[All Fields] OR "embolisations"[All Fields] OR "embolise"[All Fields] OR "embolised"[All Fields] OR "embolising"[All Fields] OR "embolism"[MeSH Terms] OR "embolism"[All Fields] OR "embolic"[All Fields] OR "embolisms"[All Fields] OR "embolization, therapeutic"[MeSH Terms] OR ("embolization"[All Fields] AND "therapeutic"[All Fields]) OR "therapeutic embolization"[All Fields] OR "embolisation"[All Fields] OR "embolization"[All Fields] OR "embolizations"[All Fields] OR "embolize"[All Fields] OR "embolized"[All Fields] OR "embolizes"[All Fields] OR "embolizing"[All Fields] OR (("pelvics"[All Fields] OR "pelvis"[MeSH Terms] OR "pelvis"[All Fields] OR "pelvic"[All Fields]) AND ("arterialization"[All Fields] OR "arterializations"[All Fields] OR "arterialize"[All Fields] OR "arterialized"[All Fields] OR "arterializing"[All Fields] OR "arterially"[All Fields] OR "arterials"[All Fields] OR "arterie"[All Fields] OR "arteries"[MeSH Terms] OR "arteries"[All Fields] OR "arterial"[All Fields] OR "arteris"[All Fields] OR "artery"[All Fields] OR "arterious"[All Fields] OR "artery s"[All	278	04:04:44

				Fields] OR "arterys"[All Fields]) AND ("embol"[All Fields] OR "embolics"[All Fields] OR "embolisations"[All Fields] OR "embolise"[All Fields] OR "embolised"[All Fields] OR "embolising"[All Fields] OR "embolism"[MeSH Terms] OR "embolism"[All Fields] OR "embolic"[All Fields] OR "embolisms"[All Fields] OR "embolization, therapeutic"[MeSH Terms] OR ("embolization"[All Fields] AND "therapeutic"[All Fields]) OR "therapeutic embolization"[All Fields] OR "embolisation"[All Fields] OR "embolization"[All Fields] OR "embolizations"[All Fields] OR "embolize"[All Fields] OR "embolized"[All Fields] OR "embolizes"[All Fields] OR "embolizing"[All Fields])) OR ("uterine artery embolization"[MeSH Terms] OR ("uterine"[All Fields] AND "artery"[All Fields] AND "embolization"[All Fields]) OR "uterine artery embolization"[All Fields]) OR "UAE"[All Fields] OR ("transcatheter"[All Fields] AND ("embol"[All Fields] OR "embolics"[All Fields] OR "embolisations"[All Fields] OR "embolise"[All Fields] OR "embolised"[All Fields] OR "embolising"[All Fields] OR "embolism"[MeSH Terms] OR "embolism"[All Fields] OR "embolic"[All Fields] OR "embolisms"[All Fields] OR "embolization, therapeutic"[MeSH Terms] OR ("embolization"[All Fields] AND "therapeutic"[All Fields]) OR "therapeutic embolization"[All Fields] OR "embolisation"[All Fields] OR "embolization"[All Fields] OR "embolizations"[All Fields] OR "embolize"[All Fields] OR "embolized"[All Fields] OR "embolizes"[All Fields] OR "embolizing"[All Fields])) OR ("chem educ"[Journal] OR "tce"[All Fields]))		
**16**	#10 OR #11 OR #12 OR #13 OR #14 OR #15			"embol"[All Fields] OR "embolics"[All Fields] OR "embolisations"[All Fields] OR "embolise"[All Fields] OR "embolised"[All Fields] OR "embolising"[All Fields] OR "embolism"[MeSH Terms] OR "embolism"[All Fields] OR "embolic"[All Fields] OR "embolisms"[All Fields] OR "embolization, therapeutic"[MeSH Terms] OR ("embolization"[All Fields] AND "therapeutic"[All Fields]) OR "therapeutic embolization"[All Fields] OR "embolisation"[All Fields] OR "embolization"[All Fields] OR "embolizations"[All Fields] OR "embolize"[All Fields] OR "embolized"[All Fields] OR "embolizes"[All Fields] OR "embolizing"[All Fields] OR (("pelvics"[All Fields] OR "pelvis"[MeSH Terms] OR "pelvis"[All Fields] OR "pelvic"[All Fields]) AND ("arterialization"[All Fields] OR "arterializations"[All Fields] OR "arterialize"[All Fields] OR "arterialized"[All Fields] OR "arterializing"[All Fields] OR "arterially"[All Fields] OR "arterials"[All Fields] OR "arterie"[All Fields] OR "arteries"[MeSH Terms] OR "arteries"[All Fields] OR "arterial"[All Fields] OR "arteris"[All Fields] OR "artery"[All Fields] OR "arterious"[All Fields] OR "artery s"[All Fields] OR "arterys"[All Fields]) AND ("embol"[All Fields] OR "embolics"[All Fields] OR "embolisations"[All Fields] OR "embolise"[All Fields] OR "embolised"[All Fields] OR "embolising"[All Fields] OR "embolism"[MeSH Terms] OR "embolism"[All Fields] OR "embolic"[All Fields] OR "embolisms"[All Fields] OR "embolization, therapeutic"[MeSH Terms] OR ("embolization"[All Fields] AND "therapeutic"[All Fields]) OR "therapeutic embolization"[All Fields] OR "embolisation"[All Fields] OR "embolization"[All Fields] OR "embolizations"[All Fields] OR "embolize"[All Fields] OR "embolized"[All Fields] OR "embolizes"[All Fields] OR "embolizing"[All Fields])) OR ("uterine artery embolization"[MeSH Terms] OR ("uterine"[All Fields] AND "artery"[All Fields] AND "embolization"[All Fields]) OR "uterine artery embolization"[All Fields]) OR "UAE"[All Fields] OR ("transcatheter"[All Fields] AND ("embol"[All Fields] OR "embolics"[All Fields] OR "embolisations"[All Fields] OR "embolise"[All Fields] OR "embolised"[All Fields] OR "embolising"[All Fields] OR "embolism"[MeSH Terms] OR "embolism"[All Fields] OR "embolic"[All Fields] OR "embolisms"[All Fields] OR "embolization, therapeutic"[MeSH Terms] OR ("embolization"[All Fields] AND "therapeutic"[All Fields]) OR "therapeutic embolization"[All Fields] OR "embolisation"[All Fields] OR "embolization"[All Fields] OR "embolizations"[All Fields] OR	191,575	04:04:11

				"embolize"[All Fields] OR "embolized"[All Fields] OR "embolizes"[All Fields] OR "embolizing"[All Fields])) OR ("chem educ"[Journal] OR "tce"[All Fields])		
15	TCE			"chem educ"[Journal] OR "tce"[All Fields]	3,779	04:03:05
14	transcatheter embolization			"transcatheter"[All Fields] AND ("embol"[All Fields] OR "embolics"[All Fields] OR "embolisations"[All Fields] OR "embolise"[All Fields] OR "embolised"[All Fields] OR "embolising"[All Fields] OR "embolism"[MeSH Terms] OR "embolism"[All Fields] OR "embolic"[All Fields] OR "embolisms"[All Fields] OR "embolization, therapeutic"[MeSH Terms] OR ("embolization"[All Fields] AND "therapeutic"[All Fields]) OR "therapeutic embolization"[All Fields] OR "embolisation"[All Fields] OR "embolization"[All Fields] OR "embolizations"[All Fields] OR "embolize"[All Fields] OR "embolized"[All Fields] OR "embolizes"[All Fields] OR "embolizing"[All Fields])	8,752	04:02:49
13	UAE			"UAE"[All Fields]	10,148	04:02:28
12	Uterine artery embolization			"uterine artery embolization"[MeSH Terms] OR ("uterine"[All Fields] AND "artery"[All Fields] AND "embolization"[All Fields]) OR "uterine artery embolization"[All Fields]	2,607	04:02:11
11	Pelvic arterial embolization			("pelvics"[All Fields] OR "pelvis"[MeSH Terms] OR "pelvis"[All Fields] OR "pelvic"[All Fields]) AND ("arterialization"[All Fields] OR "arterializations"[All Fields] OR "arterialize"[All Fields] OR "arterialized"[All Fields] OR "arterializing"[All Fields] OR "arterially"[All Fields] OR "arterials"[All Fields] OR "arterie"[All Fields] OR "arteries"[MeSH Terms] OR "arteries"[All Fields] OR "arterial"[All Fields] OR "arteris"[All Fields] OR "artery"[All Fields] OR "arterious"[All Fields] OR "artery s"[All Fields] OR "arterys"[All Fields]) AND ("embol"[All Fields] OR "embolics"[All Fields] OR "embolisations"[All Fields] OR "embolise"[All Fields] OR "embolised"[All Fields] OR "embolising"[All Fields] OR "embolism"[MeSH Terms] OR "embolism"[All Fields] OR "embolic"[All Fields] OR "embolisms"[All Fields] OR "embolization, therapeutic"[MeSH Terms] OR ("embolization"[All Fields] AND "therapeutic"[All Fields]) OR "therapeutic embolization"[All Fields] OR "embolisation"[All Fields] OR "embolization"[All Fields] OR "embolizations"[All Fields] OR "embolize"[All Fields] OR "embolized"[All Fields] OR "embolizes"[All Fields] OR "embolizing"[All Fields])	1,912	04:01:55
10	Embolization			"embol"[All Fields] OR "embolics"[All Fields] OR "embolisations"[All Fields] OR "embolise"[All Fields] OR "embolised"[All Fields] OR "embolising"[All Fields] OR "embolism"[MeSH Terms] OR "embolism"[All Fields] OR "embolic"[All Fields] OR "embolisms"[All Fields] OR "embolization, therapeutic"[MeSH Terms] OR ("embolization"[All Fields] AND "therapeutic"[All Fields]) OR "therapeutic embolization"[All Fields] OR "embolisation"[All Fields] OR "embolization"[All Fields] OR "embolizations"[All Fields] OR "embolize"[All Fields] OR "embolized"[All Fields] OR "embolizes"[All Fields] OR "embolizing"[All Fields]	178,650	04:01:29
9	#7 AND #8			("uterus"[MeSH Terms] OR "uterus"[All Fields] OR "uteri"[All Fields] OR ("uterine artery"[MeSH Terms] OR ("uterine"[All Fields] AND "artery"[All Fields]) OR "uterine artery"[All Fields]) OR ("uterine haemorrhage"[All Fields] OR "uterine hemorrhage"[MeSH Terms] OR ("uterine"[All Fields] AND "hemorrhage"[All Fields]) OR "uterine hemorrhage"[All Fields])) AND ("arteriovenous malformations"[MeSH Terms] OR ("arteriovenous"[All Fields] AND "malformations"[All Fields]) OR "arteriovenous malformations"[All Fields] OR ("arteriovenous"[All Fields] AND "malformation"[All Fields]) OR "arteriovenous malformation"[All Fields] OR ("arteriovenous fistula"[MeSH Terms] OR ("arteriovenous"[All Fields] AND	474	04:01:13

				"fistula"[All Fields]) OR "arteriovenous fistula"[All Fields]) OR "AVM"[All Fields])		
8	#4 OR #5 OR #6			"arteriovenous malformations"[MeSH Terms] OR ("arteriovenous"[All Fields] AND "malformations"[All Fields]) OR "arteriovenous malformations"[All Fields] OR ("arteriovenous"[All Fields] AND "malformation"[All Fields]) OR "arteriovenous malformation"[All Fields] OR ("arteriovenous fistula"[MeSH Terms] OR ("arteriovenous"[All Fields] AND "fistula"[All Fields]) OR "arteriovenous fistula"[All Fields]) OR "AVM"[All Fields]	46,275	04:00:47
7	#1 OR #2 OR #3			"uterus"[MeSH Terms] OR "uterus"[All Fields] OR "uteri"[All Fields] OR ("uterine artery"[MeSH Terms] OR ("uterine"[All Fields] AND "artery"[All Fields]) OR "uterine artery"[All Fields]) OR ("uterine haemorrhage"[All Fields] OR "uterine hemorrhage"[MeSH Terms] OR ("uterine"[All Fields] AND "hemorrhage"[All Fields]) OR "uterine hemorrhage"[All Fields])	187,956	03:59:34
6	AVM			"AVM"[All Fields]	6,002	03:58:45
5	arteriovenous fistula			"arteriovenous fistula"[MeSH Terms] OR ("arteriovenous"[All Fields] AND "fistula"[All Fields]) OR "arteriovenous fistula"[All Fields]	25,607	03:58:34
4	arteriovenous malformation			"arteriovenous malformations"[MeSH Terms] OR ("arteriovenous"[All Fields] AND "malformations"[All Fields]) OR "arteriovenous malformations"[All Fields] OR ("arteriovenous"[All Fields] AND "malformation"[All Fields]) OR "arteriovenous malformation"[All Fields]	37,586	03:58:22
3	Uterine Hemorrhage			"uterine haemorrhage"[All Fields] OR "uterine hemorrhage"[MeSH Terms] OR ("uterine"[All Fields] AND "hemorrhage"[All Fields]) OR "uterine hemorrhage"[All Fields]	25,640	03:58:09
2	Uterine Artery			"uterine artery"[MeSH Terms] OR ("uterine"[All Fields] AND "artery"[All Fields]) OR "uterine artery"[All Fields]	9,151	03:58:00
1	Uterus			"uterus"[MeSH Terms] OR "uterus"[All Fields] OR "uteri"[All Fields]	164,707	03:57:49

Supplementary S2: Risk of bias score guide and Risk of bias summary table

Risk of Bias score guide:

Domain	Question	Relevant points	Notes
Selection	Does the patient(s) represent(s) the whole experience of the investigator (center) or is the selection method unclear to the extent that other patients with similar presentation may not have been reported?	/1	1 point if author makes a statement giving an indication if this is the entire experience with medical management at their center
Ascertainment	Was the exposure adequately ascertained?<br>Was the outcome adequately ascertained?	/1<br>/1	Exposure: 1 point if they made a diagnosis of AVM based on their own criteria, or any US

			findings of AVM are discussed<br><br>Outcome: 1 point if any outcome (future complications, clinical course, fertility) is discussed
Causality	Was follow-up long enough for outcomes to occur?	/2	1 point if article discusses some of post-treatment course, with at least one outcome but no final follow up to resolution or treatment failure<br><br>2 points if they discuss to the point of imaging resolution or treatment failure
Reporting	Is the case(s) described with sufficient details to allow other investigators to replicate the research or to allow practitioners make inferences related to their own practice?	/2	1 point for patient selection – information on imaging/ patient factors detailed enough to allow for selection of a similar patient to attempt medical management on<br><br>1 point for treatment details – discuss at least two of the following: agent, dose, duration

ROB Summary table

Case report studies:

Author	Year of publication	Risk of bias score (/7)	Selection (1)	Ascertainment (2)	Causality (2)	Reporting (2)
Youssef A et al. (1)	2020	2	0	1	1	0
Yokomine D et al. (2)	2009	6	0	2	2	2
Yela DA et al (3)	2014	3	0	1	2	0

Woodhams R et al. (4)	2014	3	0	1	1	1
Wijesekera NT et al. (5)	2009	6	0	2	2	2
Wald DA et al. (6)	2002	3	0	1	1	1
Vandenbroucke L et al. (7)	2011	2	0	1	1	0
Tullius TG Jr et al. (8)	2015	6	0	2	2	2
Timor-Tritsch IE et al. (9)	2016	4	0	1	2	1
Takeda A et al. (10)	2009	6	0	2	2	2
Stiepel HR 3rd et al. (11)	2021	6	0	2	2	2
Singh N et al. (12)	2014	3	0	1	2	0
Singh C et al. (13)	2013	6	0	2	2	2
Silva ACBD et al. (14)	2021	6	0	2	2	2
Seo KJ et al. (15)	2013	4	0	2	2	0
Scribner D et al. (16)	2016	2	0	1	1	0
Rygh AB et al. (17)	2009	6	0	2	2	2
Rubod C et al. (18)	2005	2	0	1	1	0
Rebarber A et al. (19)	2009	6	0	2	2	2
Rangarajan RD et al. (20)	2007	6	0	2	2	2
Rampersad F et al. (21)	2020	6	0	2	2	2
Pohlan J et al. (22)	2021	6	0	2	2	2
Peitsidis P et al. (23)	2011	6	0	2	2	2
Patton EW et al. (24)	2014	6	0	2	2	2
Patel S et al. (25)	2009	6	0	2	2	2
Ore RM et al. (26)	2015	5	0	2	2	1
Oh CH et al. (27)	2021	6	0	2	2	2
Sato E et al. (28)	2015	4	0	1	2	1
Morita R et al. (29)	2021	6	0	2	2	2
Molvi SN et al. (30)	2011	5	0	2	2	1
Marshall A et al. (31)	2015	5	0	2	1	2
Lui MW et al. (32)	2013	1	0	1	0	0
Lokossou MS et al. (33)	2021	3	0	1	1	1
Lipari CW et al. (34)	2005	5	0	2	2	1
Lin CJ et al. (35)	2014	4	0	1	2	1

Lin AC et al. (36)	2007	3	0	1	1	1
Lehrman ED et al. (37)	2017	3	0	1	1	1
Lebreton C et al (38)	2020	4	0	1	2	1
Lalitha N et al. (39)	2014	2	0	1	1	0
Kurda D et al. (40)	2019	6	0	2	2	2
Kondo W et al. (41)	2016	4	0	1	2	1
Kim TH et al. (42)	2010	5	1	1	1	1
Khan S et al. (43)	2019	4	0	1	2	1
Kemal Harzif A et al. (44)	2019	5	1	1	2	1
Kelly SM et al. (45)	2003	6	0	2	2	2
Karadag B et al. (46)	2016	4	0	1	2	1
Kanenishi K et al. (47)	2012	5	0	2	2	1
Javed A et al. (48)	2018	5	0	1	2	2
Imankulova B et al. (49)	2018	3	0	1	1	0
Huynh C et al. (50)	2020	5	0	2	2	1
Hernández-Escobar CE et al. (51)	2016	4	0	1	2	1
Hashim H et al. (52)	2013	6	0	2	2	2
Hasegawa A et al. (53)	2012	6	0	2	2	2
Hammad R et al. (54)	2020	6	1	2	2	1
Halperin et al. (55)	2007	3	0	1	1	1
Guo N et al. (56)	2010	0	0	0	0	0
Gulati MS et al. (57)	2000	2	0	0	1	1
Griffin DW et al. (58)	2009	6	0	2	2	2
Giurazza F et al. (59)	2017	5	0	2	2	1
Gingold JA et al. (60)	2020	2	0	1	1	0
Ghosh A at al. (61)	2006	4	0	1	2	1
Gallagher N et al. (62)	2020	6	0	2	2	2

Soeda S et al. (63)	2013	3	0	1	1	1
Evans A et al. (64)	2017	5	0	2	1	2
Elia G et al. (65)	2001	6	0	2	2	2
El Agwany AS et al. (66)	2018	2	0	1	1	0
Dimassi K et al. (67)	2018	6	0	2	2	2
Clarke MJ et al. (68)	2003	4	0	1	2	1
Chughtai F et al. (69)	2020	2	0	1	1	0
Chittawar PB et al. (70)	2013	6	0	2	2	2
Chien SC et al. (71)	2007	6	0	2	2	2
Chen LK et al. (72)	2016	6	0	2	2	2
Chen Y at al. (73)	2013	4	0	1	2	1
Chatra P et al. (74)	2021	3	0	1	2	0
Chang FW et al. (75)	2004	6	0	2	2	2
Chan CC et al. (76)	2003	6	0	2	2	2
Castillo MS et al. (77)	2007	5	0	2	2	1
Buntrock A et al. (78)	2021	4	0	2	1	1
Delplanque S et al. (79)	2018	4	0	1	1	2
Alessandrino F et al. (80)	2013	5	0	1	2	2
Ahn HY et al. (81)	2005	5	0	2	2	1
Agarwal N et al. (82)	2017	5	0	2	2	1
TOTAL (medium)		4,5	0,04	1,5	1,7	1,2

<u>**Case series studies:**</u>

**Author**	**Year of publication**	**Risk of bias score (/7)**	**Selection (1)**	**Ascertainment (2)**	**Causality (2)**	**Reporting (2)**
Zhu YP et al. (83)	2013	7	1	2	2	2
Yang JJ et al. (84)	2005	5	1	2	2	0
Wang Z et al. (85)	2012	3	1	1	1	0
Vaknin Z et al. (86)	2011	6	1	2	2	1
Picel AC et al. (87)	2016	6	1	2	2	1

O'Brien P et al. (88)	2006	5	1	1	2	1
McGrath S et al. (89)	2012	6	1	2	2	1
Kim T et al. (90)	2014	5	0	2	2	1
Hong W et al. (91)	2020	6	1	2	2	1
Gorsi U et al. (92)	2020	3	0	1	1	1
Ghai S et al. (93)	2003	5	1	1	1	2
Delplanque S et al. (94)	2018	6	1	2	1	2
Barral PA et al. (95)	2016	5	0	2	2	1
TOTAL (medium)		5,2	0,8	1,7	1,7	1,1

REFERENCES:

1.Youssef A, Brunelli E, Modestino F. Three-dimensional color Doppler before and after embolization of postpartum-acquired enhanced myometrial vascularity/arteriovenous malformation. Am J Obstet Gynecol. 2020 Dec;223(6):925-928. doi: 10.1016/j.ajog.2020.05.002. Epub 2020 May 7. PMID: 32387322.

2.Yokomine D, Yoshinaga M, Baba Y, Matsuo T, Iguro Y, Nakajo M, Douchi T. Successful management of uterine arteriovenous malformation by ligation of feeding artery after unsuccessful uterine artery embolization. J Obstet Gynaecol Res. 2009 Feb;35(1):183-8. doi: 10.1111/j.1447-0756.2008.00875.x. PMID: 19215570.

3.Yela DA, Yoneda J, Brasil L. Uterine arteriovenous malformation after gestational trophoblastic disease: a report of two cases. J Reprod Med. 2014 Jul-Aug;59(7-8):417-20. PMID: 25098034.

4. R, Ogasawara G, Ishida K, Fujii K, Yamane T, Nishimaki H, Matsunaga K, Inoue Y. Successful treatment of acquired uterine arterial venous malformation using N-butyl-2-cyanoacrylate under balloon occlusion. Acta Radiol Short Rep. 2014 Sep 2;3(8):2047981614545910. doi: 10.1177/2047981614545910. PMID: 25346850; PMCID: PMC4207280.

5.Wijesekera NT, Padley SP, Kazmi F, Davies CL, McCall JM. Embolization of uterine arteriovenous malformations associated with cyanotic congenital heart disease. Cardiovasc Intervent Radiol. 2009 Sep;32(5):1075-9. doi: 10.1007/s00270-009-9573-z. Epub 2009 May 7. PMID: 19730767.

6.Wald DA. Postpartum hemorrhage resulting from uterine artery pseudoaneurysm. J Emerg Med. 2003 Jul;25(1):57-60. doi: 10.1016/s0736-4679(03)00098-2. PMID: 12865110.

7.Vandenbroucke L, Morcel K, Bruneau B, Moquet PY, Bauville E, Levêque J, Lavoue V. Malformations artérioveineuses endo-utérines acquises [Acquired uterine arteriovenous

malformations]. Gynecol Obstet Fertil. 2011 Jul-Aug;39(7-8):469-72. French. doi: 10.1016/j.gyobfe.2011.05.008. Epub 2011 Jul 12. PMID: 21752685.

8.Tullius TG Jr, Ross JR, Flores M, Ghaleb M, Kupesic Plavsic S. Use of three-dimensional power Doppler sonography in the diagnosis of uterine arteriovenous malformation and follow-up after uterine artery embolization: Case report and brief review of literature. J Clin Ultrasound. 2015 Jun;43(5):327-34. doi: 10.1002/jcu.22210. Epub 2014 Jul 18. PMID: 25042165.

9.Timor-Tritsch IE, Haynes MC, Monteagudo A, Khatib N, Kovács S. Ultrasound diagnosis and management of acquired uterine enhanced myometrial vascularity/arteriovenous malformations. Am J Obstet Gynecol. 2016 Jun;214(6):731.e1-731.e10. doi: 10.1016/j.ajog.2015.12.024. Epub 2016 Feb 9. PMID: 26873276.

10.Takeda A, Koyama K, Imoto S, Mori M, Sakai K, Nakamura H. Progressive formation of uterine arteriovenous fistula after laparoscopic-assisted myomectomy. Arch Gynecol Obstet. 2009 Oct;280(4):663-7. doi: 10.1007/s00404-009-0981-8. Epub 2009 Feb 18. PMID: 19224230.

11.Stiepel HR 3rd, Burke CT, Stewart JK. Embolization of uterine arteriovenous malformation causing postpartum hemorrhage using n-butyl cyanoacrylate: A case report. Radiol Case Rep. 2021 Mar 19;16(5):1188-1190. doi: 10.1016/j.radcr.2021.02.053. PMID: 33777284; PMCID: PMC7985274.

12.Singh N, Tripathi R, Mala YM, Tyagi S, Tyagi S, Singh C. Varied presentation of uterine arteriovenous malformations and their management by uterine artery embolisation. J Obstet Gynaecol. 2014 Jan;34(1):104-6. doi: 10.3109/01443615.2013.816666. PMID: 24359071.

13.Singh C, Gupta M, Tripathi R, Tyagi S. Successful use of transcatheter embolisation in an emergent life-threatening situation of bleeding from uterine arteriovenous malformation. BMJ Case Rep. 2013 Apr 18;2013:bcr2013008730. doi: 10.1136/bcr-2013-008730. PMID: 23605826; PMCID: PMC3645646.

14.Silva ACBD, Passos JP, Signorini Filho RC, Braga A, Mattar R, Sun SY. Uterine Rescue in High-Risk Gestational Trophoblastic Neoplasia Treated with EMA-CO by Uterine Arteries Embolization due to Arteriovenous Malformations. Rev Bras Ginecol Obstet. 2021 Apr;43(4):323-328. English. doi: 10.1055/s-0041-1725054. Epub 2021 May 12. PMID: 33979893

15.Seo KJ, Kim J, Sohn IS, Kwon HS, Park SW, Hwang HS. Failed transarterial embolization of subserosal uterine arteriovenous malformation. Obstet Gynecol Sci. 2013 Sep;56(5):333-7. doi: 10.5468/ogs.2013.56.5.333. Epub 2013 Sep 14. PMID: 24328024; PMCID: PMC3784124.

16.Scribner D, Fraser R. Diagnosis of Acquired Uterine Arteriovenous Malformation by Doppler Ultrasound. J Emerg Med. 2016 Aug;51(2):168-71. doi: 10.1016/j.jemermed.2016.04.028. Epub 2016 May 31. PMID: 27260690.

17.Rygh AB, Greve OJ, Fjetland L, Berland JM, Eggebø TM. Arteriovenous malformation as a consequence of a scar pregnancy. Acta Obstet Gynecol Scand. 2009;88(7):853-5. doi: 10.1080/00016340902971466. PMID: 19452327.

18.Rubod C, Mubiayi N, Robert Y, Vinatier D. Malformation artérioveineuse utérine. Une cause rare de métrorragies récidivantes [Uterine arteriovenous malformation. A rare cause of recurrent metrorrhagia]. Gynecol Obstet Fertil. 2005 Jul-Aug;33(7-8):511-3. French. doi: 10.1016/j.gyobfe.2005.05.018. PMID: 16005663.

19.Rebarber A, Fox NS, Eckstein DA, Lookstein RA, Saltzman DH. Successful bilateral uterine artery embolization during an ongoing pregnancy. Obstet Gynecol. 2009 Feb;113(2 Pt 2):554-556. doi: 10.1097/AOG.0b013e318193bfdf. PMID: 19155955.

20.Rangarajan RD, Moloney JC, Anderson HJ. Diagnosis and nonsurgical management of uterine arteriovenous malformation. Cardiovasc Intervent Radiol. 2007 Nov-Dec;30(6):1267-70. doi: 10.1007/s00270-007-9100-z. PMID: 17587081.

21.Rampersad F, Narine S, Rampersad D, Diljohn J, Ali R. Uterine arteriovenous malformation mimicking retained products Of conception - treated with embolization. Radiol Case Rep. 2020 Sep 2;15(11):2076-2079. doi: 10.1016/j.radcr.2020.08.048. PMID: 32944104; PMCID: PMC7481512.

22.Pohlan J, Hinkson L, Wickmann U, Henrich W, Althoff CE. Pseudo aneurysm of the uterine artery with arteriovenous fistula after cesarean section: A rare but sinister cause of delayed postpartum hemorrhage. J Clin Ultrasound. 2021 Mar;49(3):265-268. doi: 10.1002/jcu.22890. Epub 2020 Jun 29. PMID: 32602168.

23.Peitsidis P, Manolakos E, Tsekoura V, Kreienberg R, Schwentner L. Uterine arteriovenous malformations induced after diagnostic curettage: a systematic review. Arch Gynecol Obstet. 2011 Nov;284(5):1137-51. doi: 10.1007/s00404-011-2067-7. Epub 2011 Sep 9. PMID: 21904854.

24.Patton EW, Moy I, Milad MP, Vogezang R. Fertility-preserving management of a uterine arteriovenous malformation: a case report of uterine artery embolization (UAE) followed by laparoscopic resection. J Minim Invasive Gynecol. 2015 Jan;22(1):137-41. doi: 10.1016/j.jmig.2014.07.016. Epub 2014 Aug 10. PMID: 25117839.

25.Patel S, Potti S, Jaspan D, Dandolu V. Embolization of uterine arteriovenous malformation for treatment of menorrhagia. Arch Gynecol Obstet. 2009 Feb;279(2):229-32. doi: 10.1007/s00404-008-0684-6. Epub 2008 Jul 30. PMID: 18665382.

26.Ore RM, Lynch D, Rumsey C. Uterine arteriovenous malformation, images, and management. Mil Med. 2015 Jan;180(1):e177-80. doi: 10.7205/MILMED-D-14-00145. PMID: 25562882.

27.Oh CH, Kim Y, Cho BS, Yi KS. Successful transcatheter arterial embolization for massive hemorrhage from acquired uterine arteriovenous malformation which occurred as a complication

of hysterectomy: A case report. Medicine (Baltimore). 2021 Jan 15;100(2):e24052. doi: 10.1097/MD.0000000000024052. PMID: 33466159; PMCID: PMC7808482.

28.Sato E, Nakayama K, Nakamura K, Ishikawa M, Katagiri H, Kyo S. A case with life-threatening uterine bleeding due to postmenopausal uterine arteriovenous malformation. BMC Womens Health. 2015;15:10. doi: 10.1186/s12905-015-0163-8. Epub 2015 Feb 17. PMID: 25783637; PMCID: PMC4348402.

29.Morita R, Abo D, Kinota N, Soyama T, Takahashi B, Yoshino Y, Tsuneta S, Kudo K. Successful transvenous embbolization for type II uterine arteriovenous malformation: A case report. Radiol Case Rep. 2021 Jun 8;16(8):2007-2011. doi: 10.1016/j.radcr.2021.05.013. PMID: 34158882; PMCID: PMC8203566.

30.Molvi SN, Dash K, Rastogi H, Khanna SB. Transcatheter embolization of uterine arteriovenous malformation: report of 2 cases and review of literature. J Minim Invasive Gynecol. 2011 Nov-Dec;18(6):812-9. doi: 10.1016/j.jmig.2011.07.007. PMID: 22024270.

31.Marshall A, Patel M, Eghbalieh N, Weidenhaft M, Hanemann C, Neitzschman H. Radiology Case of the Month:Diagnosis and Treatment of an Acquired Uterine Arteriovenous Malformation in a 26-Year-Old Woman presenting with Vaginal Bleeding. J La State Med Soc. 2015 Jul-Aug;167(4):198-201. Epub 2015 Aug 15. PMID: 27159516.

32.Lui MW, Shek NW, Li RH, Chu F, Pun TC. Management of heterotopic cesarean scar pregnancy by repeated transvaginal ultrasonographic-guided aspiration with successful preservation of normal intrauterine pregnancy and complicated by arteriovenous malformation. Eur J Obstet Gynecol Reprod Biol. 2014 Apr;175:209-10. doi: 10.1016/j.ejogrb.2013.12.042. Epub 2014 Jan 13. PMID: 24491276.

33.Lokossou MS, Akouala G, Aganahi A, Vodouhe M, Lokossou AL, Tramier A, Ayhan G, Janky E. Malformation artério-veineuse utérine: à propos de deux cas au Centre Hospitalier Universitaire de la Guadeloupe [Uterine arteriovenous malformation: about two cases at the University Hospital of Guadeloupe]. Pan Afr Med J. 2021 Mar 24;38:307. French. doi: 10.11604/pamj.2021.38.307.24924. PMID: 34178225; PMCID: PMC8197057.

34.Lipari CW, Badawy SZ. Arteriovenous malformation in a bicornuate uterus leading to recurrent severe uterine bleeding: a case report. J Reprod Med. 2005 Jan;50(1):57-60. PMID: 15730176.

35.Lin CJ, Huang LW, Lin YH, Hwang JL, Seow KM. Interstitial ectopic pregnancy complicated by uterine arteriovenous malformations treated with unilateral transarterial embolization. Taiwan J Obstet Gynecol. 2014 Jun;53(2):279-81. doi: 10.1016/j.tjog.2013.11.004. PMID: 25017288.

36.Lin AC, Hung YC, Huang LC, Chiu TH, Ho M. Successful treatment of uterine arteriovenous malformation with percutaneous embolization. Taiwan J Obstet Gynecol. 2007 Mar;46(1):60-3. doi: 10.1016/S1028-4559(08)60109-6. PMID: 17389192.

37.Lehrman ED, Heller M, Poder L, Kerlan R, Huddleston HG, Kohi MP. Transvaginal Obliteration of a Complex Uterine Arteriovenous Fistula Using Ethylene Vinyl Alcohol Copolymer. J Vasc Interv Radiol. 2017 Jun;28(6):842-843. doi: 10.1016/j.jvir.2017.02.019. PMID: 28532745.

38.Lebreton C, Deffieux X, Vieillefosse S, Maitre S, Vivanti AJ. An arterio-venous malformation related to a uterine scar defect, an unusual association. J Gynecol Obstet Hum Reprod. 2020 Jun;49(6):101733. doi: 10.1016/j.jogoh.2020.101733. Epub 2020 Mar 29. PMID: 32234561.

39.Lalitha N, Seetha P, Shanmugasundaram R, Rajendiran G. Uterine Arteriovenous Malformation: Case Series and Literature Review. J Obstet Gynaecol India. 2016 Aug;66(4):282-6. doi: 10.1007/s13224-015-0680-2. Epub 2015 Mar 17. PMID: 27382223; PMCID: PMC4912488.

40.Kurda D, Guduguntla G, Maingard J, Kok HK, Lalloo S. Precipitating hydrophobic injectable liquid (PHIL) embolic for the treatment of a uterine arteriovenous malformation: a technical report. CVIR Endovasc. 2019 May 17;2(1):17. doi: 10.1186/s42155-019-0059-z. PMID: 32026176; PMCID: PMC7224241.

41.Kondo W, Tessmann Zomer M, Erzinger FL. Uterine arteriovenous fistula after perforation during the placement of an intrauterine device - Minimally invasive treatment using uterine artery embolization. Clin Exp Obstet Gynecol. 2016;43(4):602-605. PMID: 29734558.

42.Kim TH, Lee HH. Presenting features of women with uterine arteriovenous malformations. Fertil Steril. 2010 Nov;94(6):2330.e7-10. doi: 10.1016/j.fertnstert.2010.03.031. Epub 2010 Apr 22. PMID: 20416872.

43.Khan S, Saud S, Khan I, Achakzai B. Acquired Uterine Arteriovenous Malformation Following Dilatation and Curettage Treated with Bilateral Uterine Artery Embolization: A Case Report. Cureus. 2019 Mar 13;11(3):e4250. doi: 10.7759/cureus.4250. PMID: 31131173; PMCID: PMC6516630.

44.Kemal Harzif A, Rei OG, Haloho A, Silvia M, Pratama G, Og Rei, MRepSc, Purwosunu Y, Og Mfm, Ph D, Wibawa A, Og Mfm, Sidipratomo P, Rad Ir, Ph D, Pandelaki J, Rad Ir, Ph D. Trans-arterial embolization of acquired uterine arteriovenous malformation after Cesarean section: A case series. Int J Reprod Biomed. 2019 Mar 19;17(2):135–42. doi: 10.18502/ijrm.v17i2.3991. PMID: 31435591; PMCID: PMC6693317.

45. Kelly SM, Belli AM, Campbell S. Arteriovenous malformation of the uterus associated with secondary postpartum hemorrhage. Ultrasound Obstet Gynecol. 2003 Jun;21(6):602-5. doi: 10.1002/uog.148. PMID: 12808679.

46.Karadag B, Erol O, Ozdemir O, Uysal A, Alparslan AS, Gurses C, Koroglu M. Successful Treatment of Uterine Arteriovenous Malformation due to Uterine Trauma. Case Rep Obstet Gynecol. 2016;2016:1890650. doi: 10.1155/2016/1890650. Epub 2016 Sep 6. PMID: 27699074; PMCID: PMC5028872.

47.Kanenishi K, Mashima M, Tanaka H, Nagasaka H, Toyama Y, Hata T. Transvaginal 3D HD-flow in diagnosis of uterine arteriovenous malformation. Arch Gynecol Obstet. 2012 Aug;286(2):541-4. doi: 10.1007/s00404-012-2284-8. Epub 2012 Mar 6. PMID: 22392491.

48.Javed A, Chander R, Hoodbhoy Z. Uterine Arteriovenous Malformations after Suction Evacuation of Missed Miscarriage. J Coll Physicians Surg Pak. 2018 Mar;28(3):S33-S34. doi: 10.29271/jcpsp.2018.03.S33. PMID: 29482699.

49.Imankulova B, Terzic M, Ukybassova T, Bapayeva G, Lesbekov T, Mustafinova G, Smagulov N, Shauyen F. Repeated pulmonary embolism with cardiac arrest after uterine artery embolization for uterine arteriovenous malformation: A case report and literature review. Taiwan J Obstet Gynecol. 2018 Dec;57(6):890-893. doi: 10.1016/j.tjog.2018.10.021. PMID: 30545548.

50.Huynh C, Ha TN, Hoang VT, Huynh PH. Acquired life-threatening uterine arteriovenous malformation treated by endovascular embolization. Radiol Case Rep. 2020 Nov 28;16(2):241-245. doi: 10.1016/j.radcr.2020.11.013. PMID: 33304434; PMCID: PMC7708760.

51.Hernández-Escobar CE, Carrillo-Martínez MA, Arroyo-Lemarroy T, ZamoraMorales MT, Garza-García GA, Campos-Sanmiguel E. Malformación arteriovenosa uterina como causa de hemorragia uterina súbita. Utilidad del ultrasonido Doppler de consultorio, otros métodos de imagen y tratamiento de mínima invasion [Uterine arteriovenous malformation as cause of uterine bleeding of sudden onset. Doppler ultrasound utility, other imaging methods and the minimally invasive treatment]. Ginecol Obstet Mex. 2016 Aug;84(8):535-41. Spanish. PMID: 29424516.

52.Hashim H, Nawawi O. Uterine arteriovenous malformation. Malays J Med Sci. 2013 Mar;20(2):76-80. PMID: 23983582; PMCID: PMC3744004.

53.Hasegawa A, Sasaki H, Wada-Hiraike O, Osuga Y, Yano T, Usman SM, Akahane M, Kozuma S, Taketani Y. Uterine arteriovenous fistula treated with repetitive transcatheter embolization: case report. J Minim Invasive Gynecol. 2012 Nov-Dec;19(6):780-4. doi: 10.1016/j.jmig.2012.07.004. PMID: 23084687.

54.Hammad R, Nausheen S, Malik M. A Case Series on Uterine Arteriovenous Malformations: A Life-Threatening Emergency in Young Women. Cureus. 2020 Jul 27;12(7):e9410. doi: 10.7759/cureus.9410. PMID: 32864239; PMCID: PMC7449621.

55.Halperin R, Schneider D, Maymon R, Peer A, Pansky M, Herman A. Arteriovenous malformation after uterine curettage: a report of 3 cases. J Reprod Med. 2007 May;52(5):445-9. PMID: 17583252.

56.Guo N, Liu H, Peng Z. Uterine arteriovenous fistula necessitating hysterectomy after two unsuccessful embolizations in an 18-year-old patient. Ann Vasc Surg. 2010 Aug;24(6):827.e9-11. doi: 10.1016/j.avsg.2010.02.041. Epub 2010 May 20. PMID: 20488655.

57.Gulati MS, Paul SB, Batra A, Sarma D, Dadhwal V, Nath J. Uterine arteriovenous malformations: the role of intravenous 'dual-phase' CT angiography. Clin Imaging. 2000 Jan-Feb;24(1):10-4. doi: 10.1016/s0899-7071(00)00155-8. PMID: 11120411.

58.Griffin DW, Strand EA. Arteriovenous malformation of the uterus after a midtrimester loss: a case report. J Reprod Med. 2009 May;54(5):333-6. PMID: 19517703.

59.Giurazza F, Corvino F, Paladini A, Borzelli A, Scognamiglio D, Frauenfelder G, Albano G, Sirimarco F, Niola R. Uterine Arteriovenous Fistula with Concomitant Pelvic Varicocele: Endovascular Embolization with Onyx-18®. Case Rep Vasc Med. 2017;2017:3548271. doi: 10.1155/2017/3548271. Epub 2017 Nov 22. PMID: 29359062; PMCID: PMC5735662.

60.Gingold JA, Bradley LD. Use of hysteroscopy in diagnosis and follow-up of acquired uterine enhanced myometrial vascularity. Fertil Steril. 2020 Feb;113(2):460-462. doi: 10.1016/j.fertnstert.2019.11.006. PMID: 32106997.

61.Ghosh A, Ayers KJ. Uterine arteriovenous malformation following medical termination of pregnancy: a case report. Arch Gynecol Obstet. 2006 Jul;274(4):250-1. doi: 10.1007/s00404-006-0159-6. Epub 2006 Apr 29. PMID: 16649041.

62.Gallagher N, Cincotta M, Keblawi H, Jude D, Korona M. Uterine arteriovenous malformation leading to postpartum hemorrhage: A case report. Case Rep Womens Health. 2020 Oct 3;28:e00260. doi: 10.1016/j.crwh.2020.e00260. PMID: 33088725; PMCID: PMC7559227.

63.Soeda S, Kyozuka H, Suzuki S, Yasuda S, Nomura Y, Fujimori K. Uterine artery embolization for uterine arteriovenous malformation is associated with placental abnormalities in the subsequent pregnancy: two cases report. Fukushima J Med Sci. 2014;60(1):86-90. doi: 10.5387/fms.2013-13. Epub 2014 Mar 27. PMID: 24670673.

64.Evans A, Gazaille RE 3rd, McKenzie R, Musser M, Lemming R, Curry J, Meyers W, Austin N. Acquired uterine arteriovenous fistula following dilatation and curettage: an uncommon cause of vaginal bleeding. Radiol Case Rep. 2017 Feb 21;12(2):287-291. doi: 10.1016/j.radcr.2017.01.005. PMID: 28491172; PMCID: PMC5417766.

65.Elia G, Counsell C, Singer SJ. Uterine artery malformation as a hidden cause of severe uterine bleeding. A case report. J Reprod Med. 2001 Apr;46(4):398-400. PMID: 11354845.

66.El Agwany AS, Elshafei M. Extensive uterine arteriovenous malformation with hemodynamic instability: Embolization for whole myometrium affection. Eur J Obstet Gynecol Reprod Biol. 2018 Mar;222:188-191. doi: 10.1016/j.ejogrb.2018.01.018. Epub 2018 Jan 31. PMID: 29395294.

67.Dimassi K, Ben Amor A, Halouani A, Dali N, Ben Farhat L, Triki A. Successful management of acquired uterine arteriovenous malformation. Tunis Med. 2018 Jul;96(7):445-447. PMID: 30430490.

68. Clarke MJ, Mitchell PJ. Uterine arteriovenous malformation: a rare cause of uterine bleeding. Diagnosis and treatment. Australas Radiol. 2003 Sep;47(3):302-5. doi: 10.1046/j.1440-1673.2003.01182.x. PMID: 12890254.

69.Chughtai F, Ahmed M, Alam AM. Uterine arterio-venous malformation, an uncommon life-threatening condition: a case report. J Pak Med Assoc. 2020 Mar;70(3):531-533. doi: 10.5455/JPMA.9800. PMID: 32207441.

70.Chittawar PB, Patel K, Agrawal P, Bhandari S. Hysteroscopic diagnosis and successful management of an acquired uterine arteriovenous malformation by percutaneous embolotherapy. J Midlife Health. 2013 Jan;4(1):57-9. doi: 10.4103/0976-7800.109641. PMID: 23833538; PMCID: PMC3702069.

71.Chien SC, Tseng SC, Hwa HL, Wei MC. Immediate post-partum haemorrhage caused by rupture of uterine arteriovenous malformation. Aust N Z J Obstet Gynaecol. 2007 Jun;47(3):252-4. doi: 10.1111/j.1479-828X.2007.00729.x. PMID: 17550497.

72.Chen LK, Yang BL, Chen KC, Tsai YL. Successful Transarterial Embolization of Uterine Arteriovenous Malformation: Report of Three Cases. Iran J Radiol. 2016 Jan 14;13(1):e15358. doi: 10.5812/iranjradiol.15358. PMID: 27110329; PMCID: PMC4835739.

73.Chen Y, Wang G, Xie F, Wang B, Tao G, Kong B. Embolization of uterine arteriovenous malformation. Iran J Reprod Med. 2013 Feb;11(2):159-66. PMID: 24639742; PMCID: PMC3941356.

74.Chatra P. Iatrogenic uterine injury leading to uterine arteriovenous malformation. Radiol Case Rep. 2021 Jun 10;16(8):2146-2150. doi: 10.1016/j.radcr.2021.05.014. PMID: 34168714; PMCID: PMC8207175.

75.Chang FW, Ding DC, Chen DC, Yu MH. Heavy uterine bleeding due to uterine arteriovenous malformations. Acta Obstet Gynecol Scand. 2004 Jun;83(6):599-600. doi: 10.1111/j.0001-6349.2004.00082b.x. PMID: 15144345.

76. CC, Chu F, Pun TC. Treating a recurrent uterine arteriovenous malformation with uterine artery embolization. A case report. J Reprod Med. 2003 Nov;48(11):905-7. PMID: 14686027.

77.Castillo MS, Borge MA, Pierce KL. Embolization of a traumatic uterine arteriovenous malformation. Semin Intervent Radiol. 2007 Sep;24(3):296-9. doi: 10.1055/s-2007-985737. PMID: 21326471; PMCID: PMC3036327.

78.Buntrock A, Hansen K, Peck S, Von Wald T. A Case of Uterine Arteriovenous Malformation Treated With Uterine Artery Embolization. S D Med. 2021 Jan;74(1):14-16. PMID: 33691051

79.Delplanque S, Le Lous M, Bauville E, Bruneau B, Levêque J, Lavoué V, Nyangoh Timoh K. Acquired uterine arteriovenous malformation in caesarean scar after a previous ectopic pregnancy:

A case report. Eur J Obstet Gynecol Reprod Biol. 2018 May;224:203-204. doi: 10.1016/j.ejogrb.2018.03.027. Epub 2018 Mar 28. PMID: 29628181.

80.Alessandrino F, Di Silverio E, Moramarco LP. Uterine arteriovenous malformation. J Ultrasound. 2013 Feb 23;16(1):41-4. doi: 10.1007/s40477-013-0007-z. PMID: 24046800; PMCID: PMC3774899.

81.Ahn HY, Park IY, Lee G, Kim SJ, Shin JC. Uterine arteriovenous malformation. Arch Gynecol Obstet. 2005 Feb;271(2):172-5. doi: 10.1007/s00404-003-0588-4. Epub 2004 Jul 15. PMID: 15257407.

82.Agarwal N, Chopra S, Aggarwal N, Gorsi U. Congenital Uterine Arteriovenous Malformation Presenting as Postcoital bleeding: A Rare Presentation of a Rare Clinical Condition. J Clin Imaging Sci. 2017 Feb 27;7:11. doi: 10.4103/jcis.JCIS_95_16. PMID: 28400997; PMCID: PMC5359997.

83.Zhu YP, Sun ZJ, Lang JH, Pan J. Clinical Characteristic and Management of Acquired Uterine Arteriovenous Malformation. Chin Med J (Engl). 2018 Oct 20;131(20):2489-2491. doi: 10.4103/0366-6999.243570. PMID: 30334536; PMCID: PMC6202597.

84.Yang JJ, Xiang Y, Wan XR, Yang XY. Diagnosis and management of uterine arteriovenous fistulas with massive vaginal bleeding. Int J Gynaecol Obstet. 2005 May;89(2):114-9. doi: 10.1016/j.ijgo.2004.11.037. PMID: 15847873.

85.Wang Z, Chen J, Shi H, Zhou K, Sun H, Li X, Pan J, Zhang X, Liu W, Yang N, Jin Z. Efficacy and safety of embolization in iatrogenic traumatic uterine vascular malformations. Clin Radiol. 2012 Jun;67(6):541-5. doi: 10.1016/j.crad.2011.11.002. Epub 2012 Jan 18. PMID: 22261390.

86.Vaknin Z, Sadeh-Mefpechkin D, Halperin R, Altshuler A, Amir P, Maymon R. Pregnancy-related uterine arteriovenous malformations: experience from a single medical center. Ultraschall Med. 2011 Dec;32 Suppl 2:E92-9. doi: 10.1055/s-0031-1273274. Epub 2011 May 25. PMID: 21614746.

87.Picel AC, Koo SJ, Roberts AC. Transcatheter Arterial Embolization with n-Butyl Cyanoacrylate for the Treatment of Acquired Uterine Vascular Malformations. Cardiovasc Intervent Radiol. 2016 Aug;39(8):1170-6. doi: 10.1007/s00270-016-1328-z. Epub 2016 Mar 28. PMID: 27021069.

88.O'Brien P, Neyastani A, Buckley AR, Chang SD, Legiehn GM. Uterine arteriovenous malformations: from diagnosis to treatment. J Ultrasound Med. 2006 Nov;25(11):1387-92; quiz 1394-5. doi: 10.7863/jum.2006.25.11.1387. PMID: 17060424.

89.McGrath S, Harding V, Lim AK, Burfitt N, Seckl MJ, Savage P. Embolization of uterine arteriovenous malformations in patients with gestational trophoblastic tumors: a review of patients at Charing Cross Hospital, 2000-2009. J Reprod Med. 2012 Jul-Aug;57(7-8):319-24. PMID: 22838248.

90.Kim T, Shin JH, Kim J, Yoon HK, Ko GY, Gwon DI, Yang H, Sung KB. Management of bleeding uterine arteriovenous malformation with bilateral uterine artery embolization. Yonsei Med J. 2014 Mar;55(2):367-73. doi: 10.3349/ymj.2014.55.2.367. PMID: 24532505; PMCID: PMC3936651.

91.Hong W, Wang BY, Wu ZP, Gao F, Li SD, Li XC. Systematic retrospective analysis of 13 cases of uterine arteriovenous fistula: Pathogeny, diagnosis, treatment and follow-up. J Obstet Gynaecol Res. 2020 Jul;46(7):1117-1127. doi: 10.1111/jog.14264. Epub 2020 May 4. PMID: 32367675.

92.Gorsi U, Bansal A, Chaluvashetty SB, Lal A, Kalra N, Kang M, Suri V, Sandhu MS. Interventional radiology in the management of uncommon causes of obstetric haemorrhage. Eur J Radiol. 2021 Jan;134:109415. doi: 10.1016/j.ejrad.2020.109415. Epub 2020 Nov 17. PMID: 33278733.

93.Ghai S, Rajan DK, Asch MR, Muradali D, Simons ME, TerBrugge KG. Efficacy of embolization in traumatic uterine vascular malformations. J Vasc Interv Radiol. 2003 Nov;14(11):1401-8. doi: 10.1097/01.rvi.0000096761.74047.7d. PMID: 14605105.

94.Delplanque S, Le Lous M, Proisy M, Joueidi Y, Bauville E, Rozel C, Beraud E, Bruneau B, Levêque J, Lavoué V, Nyangoh Timoh K. Fertility, Pregnancy, and Clinical Outcomes after Uterine Arteriovenous Malformation Management. J Minim Invasive Gynecol. 2019 Jan;26(1):153-161. doi: 10.1016/j.jmig.2018.05.001. Epub 2018 May 14. PMID: 29772406.

95.Barral PA, Saeed-Kilani M, Tradi F, Dabadie A, Izaaryene J, Soussan J, Bartoli JM, Vidal V. Transcatheter arterial embolization with ethylene vinyl alcohol copolymer (Onyx) for the treatment of hemorrhage due to uterine arteriovenous malformations. Diagn Interv Imaging. 2017 May;98(5):415-421. doi: 10.1016/j.diii.2016.09.003. Epub 2016 Oct 21. PMID: 27776896.

Supplementary S3 . PRISMA Flow Diagram

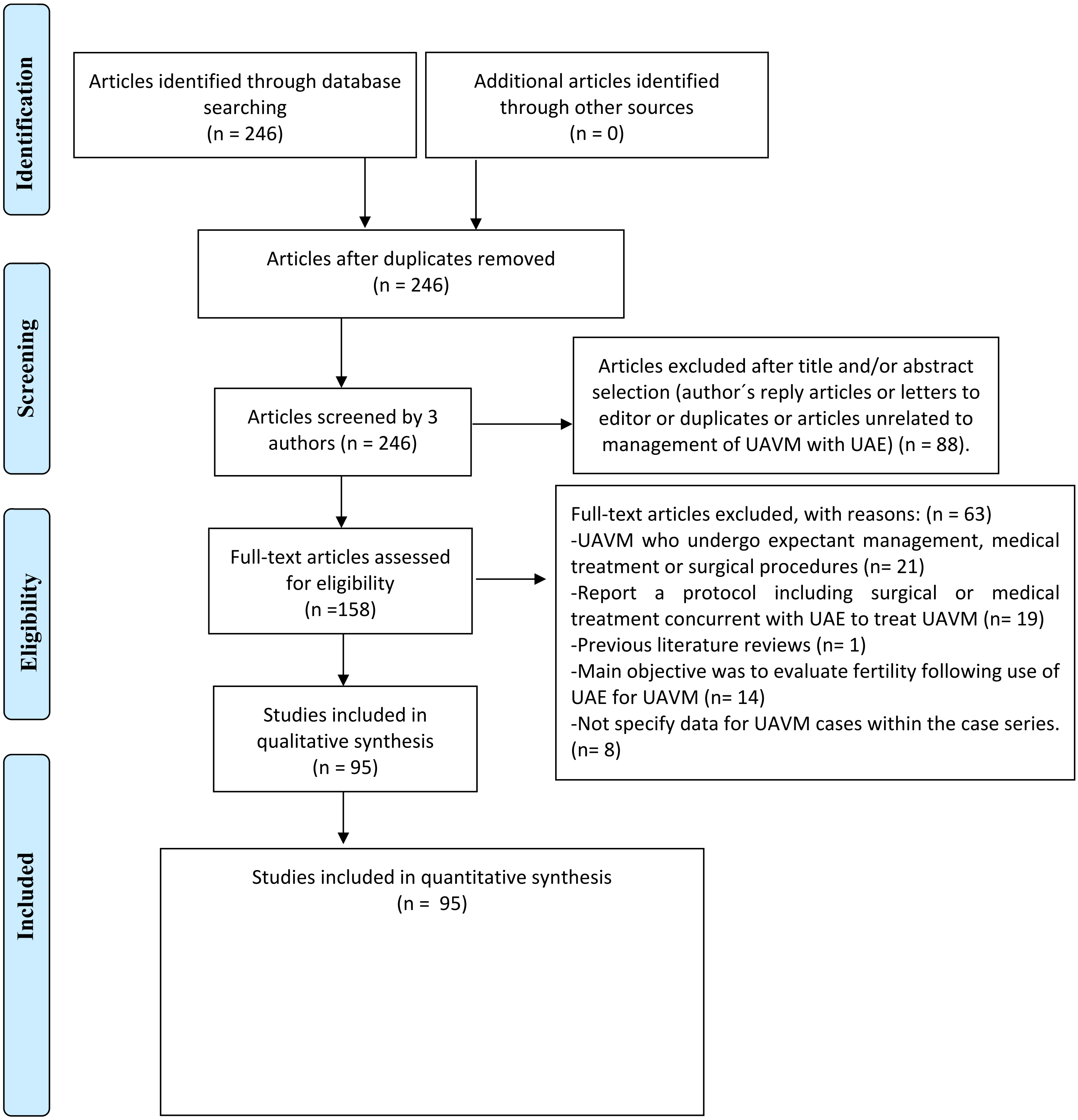

Supplement: Supplementary file 1 [file jpm-12-01098-s001.zip › jpm-1745888-supplementary.pdf]
